# Effect of Sodium Nitrate and Cysteamine on In Vitro Ruminal Fermentation, Amino Acid Metabolism and Microbiota in Buffalo

**DOI:** 10.3390/microorganisms10102038

**Published:** 2022-10-14

**Authors:** Yanxia Guo, Faiz-ul Hassan, Mengwei Li, Huade Xie, Lijuan Peng, Zhenhua Tang, Chengjian Yang

**Affiliations:** 1 Key Laboratory of Buffalo Genetics, Breeding and Reproduction Technology, Ministry of Agriculture and Guangxi Buffalo Research Institute, Chinese Academy of Agricultural Sciences, Nanning 530001, China; 2 Institute of Animal and Dairy Sciences, Faculty of Animal Husbandry, University of Agriculture, Faisalabad 38040, Pakistan

**Keywords:** nitrate, cysteamine, in vitro batch culture, methanogenesis, rumen microorganism

## Abstract

Nitrate is used as a methane inhibitor while cysteamine is considered as a growth promoter in ruminants. The present study evaluated the effect of sodium nitrate and cysteamine on methane (CH_4_) production, rumen fermentation, amino acid (AA) metabolism, and rumen microbiota in a low protein diet. Four treatments containing a 0.5 g of substrate were supplemented with 1 mg/mL sodium nitrate (SN), 100 ppm cysteamine hydrochloride (CS), and a combination of SN 1 mg/mL and CS 100 ppm (CS+SN), and a control (no additive) were applied in a completely randomized design. Each treatment group had five replicates. Two experimental runs using in vitro batch culture technique were performed for two consecutive weeks. Total gas and CH_4_ production were measured in each fermentation bottle at 3, 6, 9, 12, 24, 48, and 72 h of incubation. The results showed that SN and CS+SN reduced the production of total gas and CH_4_, increased the rumen pH, acetate, acetate to propionate ratio (A/P), and microbial protein (MCP) contents (*p* < 0.05), but decreased other volatile fatty acids (VFA) and total VFA (*p* = 0.001). The CS had no effect on CH_4_ production and rumen fermentation parameters except for increasing A/P. The CSN increased the populations of total bacteria, fungi, and methanogens but decreased the diversity and richness of rumen microorganisms. In conclusion, CS+SN exhibited a positive effect on rumen fermentation by increasing the number of fiber degrading and hydrogen-utilizing bacteria, with a desirable impact on rumen fermentation while reducing total gas and CH_4_ production.

## 1. Introduction

Rumen fermentation in ruminants leads to the production of methane (CH_4_), subsequently resulting in ~5% losses of dietary energy in addition to contributing towards greenhouse gas (GHG) loads [1]. Reducing methane production is envisioned as a potential strategy to improve the feed efficiency in ruminants and reduce greenhouse gas emissions. Different types of compounds have been tested to evaluate their efficiency to reduce CH_4_ production under in vitro and in vivo conditions while optimizing rumen fermentation. One of the major strategies to reduce methanogenesis is the use of hydrogen-consuming compounds [2]. Nitrate is a frequently used hydrogen-consuming compound to reduce methane production, which also serves as a non-protein nitrogen source for rumen microorganisms [3]. Studies have proved the utility of nitrate in low-protein diets as a non-protein nitrogen source without any toxic effects on ruminants [4]. The addition of calcium nitrate to the lamb diet resulted in a 17.3% reduction in CH_4_ emissions per kg of body weight gain and a 35.4% reduction in CH_4_ emissions per kg of dry matter intake [5]. Nitrate can inhibit methanogenesis by competing for hydrogen atoms to reduce the H_2_ availability for methane synthesis and also directly posing toxic effects on the rumen microorganisms.

In recent years, nutritionists consider amino acid contents instead of crude protein to optimize the requirements of ammonia and amino acids for efficient rumen fermentation to maximize the synthesis of microbial protein (MCP) [6]. Total dietary protein can be reduced without adverse effects on production by fulfilling the amino acid requirements of rumen microbes. Especially during the dry period, low-energy and low-protein diets can be fed to buffalo. Supplementing high-quality amino acids to the low-protein diet of dairy cows can improve nitrogen utilization while reducing nitrogen emissions. It can help to reduce the environmental pollution caused by nitrogen emissions in animal manure. Cysteine, as a sulfur-containing amino acid, is proteogenic with methionine [7]. Cysteamine is the decarboxylation product of cysteine and serves as a component of coenzyme a molecule, a bioactive substance in animals to promote body metabolism and growth. Cysteamine possesses great potential as a growth promoter in animal production owing to its diverse ability to improve feed conversion efficiency promote growth and development, regulate endocrine, alleviate stress, and enhance the lactation performance of ruminants and nitrogen utilization efficiency while reducing methane emissions [8]. Supplementation of cysteamine (0.8%) to concentrate feed resulted in an increase n the MCP content and promoted the rumen fermentation in buffalo [9]. We hypothesized that the supplementation of cysteamine in the presence of an anti-methanogenic compound (sodium nitrate) in a low-protein diet can enhance ammonia incorporation into MCP in the rumen, which might be nutritionally beneficial. Therefore, it is imperative to evaluate the synergistic effect of Cysteamine and nitrate on nitrogen and amino acid metabolism, which will help to design better nutritional interventions to improve nitrogen use efficiency and reduce CH_4_ emissions. This in vitro experiment evaluated the effect of adding sodium nitrate, cysteamine, and their mixtures in a low protein diet (90% roughage, 10% concentrate) on the cumulative gas and CH_4_ production, rumen fermentation parameters, amino acid metabolism, and rumen microbial populations.

## 2. Materials and Methods

### 2.1. Substrates and Treatments

The substrate was a low protein diet, which was composed of 90% elephant grass and 10% concentrate on a dry matter (DM) basis. Details of the chemical composition of the substrate are given in Table 1.

Four treatments containing a 0.5 g of the substrate (90% roughage and 10% concentrate mixture) were supplemented with 1 mg/mL SN (>99% purity; Baishi Chemical Reagent Co., Tianjin, China), 100 ppm CS (27% purity; Huakuoda Biology Chemical Technology Co., Shanghai, China) and a combination of 1 mg/mL SN and 100 ppm CS and a control (no additive) were applied in a completely randomized design. The control group was used to correct for sensitivity variations induced by the inocula. In addition, a blank control group was set without substrate, SN, and CS. The blank group was used to address the variations of rumen fluid used for the in vitro fermentation and to obtain the net gas production. Samples with variations above 10% were rejected. Each treatment group had five incubation bottles as replicates per run.

### 2.2. In Vitro Batch Culture

Three female buffaloes with permanent rumen fistula were selected as rumen fluid donors. These buffaloes were fed on the same ration consisting of elephant grass and concentrate *ad libitum*, which was used as a substrate for in vitro culture. Before morning feeding, the fistula cover was opened to collect the rumen contents. After collection, the rumen contents of three buffalo were mixed at a ratio of 1:1:1, blended for 10 s, squeezed, and filtered twice through two layers of gauze in the collection bottle preheated (at 39 °C) under a continuous flow of CO_2_ [10]. Two needles were inserted into the incubation bottle (180 mL) containing 0.5 g substrate accurately weighed, and rumen fluid (20 mL) and buffer solution (40 mL) were added into each incubation bottle through one of the needles [10]. The two needles were inserted to ensure the balance of air pressure inside and outside the incubation bottle and also to avoid negative pressure. The incubation bottles were continuously flushed with CO_2_ to maintain an anaerobic environment, mixed evenly, then placed in a preheated constant temperature water bath, and incubated at 39 °C for 72 h with continuous oscillation. Two experimental runs were performed for two consecutive weeks using the same experimental conditions.

### 2.3. Determination of Total Gas, Methane (CH_4_) Production and Hydrogen Balance

Gas and CH_4_ production were measured in each in vitro culture bottle at 3, 6, 9, 12, 24, 48, and 72 h of incubation. The gas production was measured with 100 mL lubricated glass syringes with a soft short tube as described previously [10]. Briefly, at each detection time point, the needle of the syringe was inserted into the incubator bottle while placing the syringe horizontally, and the gas pressure pushes the piston to move until the scale remains unchanged. Then the syringe and needle were unplugged, and the measurement was recorded. Net gas production (mL) = gas production in time period (mL) − blank average gas production in corresponding time period (mL). The cumulative total gas production in 72 h was the sum of the net gas production of the incubation bottle at each time point.

At the same time of gas measurement, the CH_4_ production was measured through gas chromatography (Agilent 7890a, Agilent Technologies, Santa Clara, CA, USA) as described previously [11]. A 10 μL sample of gas was taken from the incubation bottle and injected directly into the gas chromatograph with a manual injection needle. The chromatographic column was HP-INNOWAX (19091N-133) capillary column with a specification of 30 m × 0.25 mm × 0.25 μm. The cumulative CH_4_ production in 72 h was the sum of the actual CH_4_ production of the incubation bottle at each time point.

The hydrogen balance was calculated by using the equation developed by Demeyer [12] considering both VFA and CH_4_ production. Products such as lactate, formate, and succinate are not considered in this equation:

H_2_ produced (mol) = 2A + P + 4B + 2iV + 2V,

H_2_ utilized (mol) = 4M + 2P + 3B + V,

H_2_ Recovery (%) = H_2_ utilized/H_2_ produced × 100 = (4M + 2P + 3B + V)/(2A + P + 4B + 2iV + 2V) × 100

where: A = acetate; B = butyrate; P = propionate; M = CH_4_; iV = isovalerate; and V = valerate (net molar production).

### 2.4. Determination of Rumen Fermentation Parameters

At the end of 72 h of incubation, the incubation bottles were taken out and immediately, and cooled for 15 min by putting them into the ice water mixture to terminate the fermentation. The pH of the culture medium was measured with a pH meter (HANNA HI 8424, Shanghai Heyi Instrument Co., Ltd., Shanghai, China). About 8 mL of culture medium was used for the determination of microbial protein (MCP) content through colorimetry by using an ultraviolet-visible spectrophotometer (PE lambda 35, Shanghai Pudi Biotechnology Co., Ltd., Shanghai, China). Similarly, ammonia nitrogen (NH_3_-N) was determined by phenol sodium hypochlorite colorimetry through an ultraviolet-visible spectrophotometer at 560 nm wavelength as described previously [13]. The VFA content was determined by mixing 1 mL culture medium and 0.5 mL metaphosphoric acid (8.2%) and then centrifuging at 20,000× *g* (4 °C) for 10 min. After centrifugation, 920 µL of supernatant was added to 80 µL internal standard crotonic acid (1 mol/L). Different VFA fractions (C2, C3, C4, C5, iC4, and iC5) were measured using the GC system as described previously [11]. For the determination of dry matter digestibility (DMD), the residue and remaining liquid in the incubation bottle were filtered on the dried and weighed nylon bag, and the residue was fully washed with distilled water. The washed residue and nylon bag were dried at 105 °C to constant weight and DMD was calculated as:

DMD (%) = (1 − weight of residue after digestion/weight of substrate before digestion) × 100

### 2.5. Determination of Amino Acid Concentration

At the end of 72 h of incubation, 5 mL of the culture filtrate was mixed and hydrolyzed with 5 mL of HCl (6 mol/L) in a constant temperature oven at 110 °C for 22 h. Then, concentrations of individual amino acids were determined through liquid chromatography-tandem mass spectrometry (LC-MS/MS) analysis using a SCIEX Triple Quad 5500 LC-MS/MS System (AB SCIEX (Pvt.) Ltd., Framingham, MA, USA) as reported previously [14]. The cation exchange column was used for amino acid analysis. The column temperature was 65 °C, and the elution gradient was 100% A–100% B linear gradient. The detector was Waters 470 fluorescent detector. Finally, the percentage content of each amino acid was calculated according to the peak area of each amino acid in the chromatogram.

### 2.6. DNA Extraction and Determination of Microbial Population

The total microbial DNA in rumen fluid was extracted by the cetyltrimethylammonium bromide (CTAB) method as described previously [15]. The purity and concentration of DNA were determined by an ultramicro spectrophotometer (Nanodrop ND-2000, Beijing Xinxing Johnson Biotechnology Co., Ltd., Beijing, China). The quality of extracted DNA was checked by Nanodrop and samples with poor quality were re-extracted to get DNA of optimum quality required for quantitative real-time PCR (qRT-PCR) and high throughput sequencing.

Quantitative real-time PCR (qRT-PCR) was used to quantify the microbial populations in the rumen fluid by using methods as described in our previous study [16]. The primers used for bacteria were UniF(306) (GTGSTGCAYGGYYGTCGTCA) and UniR(309) (ACGTCRTCCMCNCCTTCCTC) [17]; for fungi, FungiF (GAGGAAGTAAAAGTCGTAACAAGGTTTC) and FungiR (CAAATTCACAAAGGGTAGGATGATT) [15]; for Protozoa, ProtozoaF (GCTTTCGWTGGTAGTGTATT) and PotozoaR (CTTGCCCTCYAATCGTWCT) [15]; for Methanogens, Met630F(501) (GGATTAGATACCCSGGTAGT) and Met803R (GTTGARTCCAATTAAACCGCA) [18]. PCR was performed using the SYBRGreen fluorescent dye in a Roche light cycler 480 RT-PCR machine (Roche, Basel, Switzerland). A 20 µL reaction volume containing 9.2 µL SYBR green mixture, 1 µL each of forward and reverse primers of respective microbial species (10 µM), and 8.0 µL nuclease-free water was used for RT-PCR. The amplification profile of RT-PCR for all primer pairs consisted of an initial denaturation for 10 min followed by 40 cycles of 95 °C for 15 s and annealing at 60 °C for 60 s. Standard curves were generated using tenfold serial dilutions of DNA from a pure culture of each microbial species after amplification through conventional PCR (95 °C for 10 s, 60 °C for 60 s for 40 cycles). The specificity of amplified products for each primer was confirmed by melting temperatures and dissociation curves after each amplification. Amplification efficiencies for each primer pair were investigated by examining the dilution series of total ruminal microbial DNA templates on the same plate in triplicate. An R^2^ value of >0.999 in the standard curve of all primers, indicated the optimum efficiency of primers. The concentration and purity of PCR products were determined by a Nanodrop spectrophotometer (NanodDrop ND-2000, Beijing Xinxing Johnson Biotechnology Co., Ltd., Beijing, China). The copy number of each standard was calculated by using the length of the PCR product and its respective DNA concentration. The copy number of each unknown sample was calculated through the association of threshold cycle (CT) values to standard curves. The results were then transformed to log10 copies/mL of the sample for further statistical analysis.

### 2.7. 16 S rDNA Gene Sequencing and Bioinformatic Analysis

High throughput (Illumina MiSeq PE300) sequencing of the 16S rRNA gene was carried out using barcoded primers for the V3–V4 region. Based on the original data obtained by the illuminamiseqtm sequencing platform, the paired reads were spliced into a sequence according to the overlapping relationship between PE Reads, and then the samples were identified and distinguished according to the barcode tag sequence and primer sequence at the beginning and end of the sequence to obtain each sample data, Finally, the quality of each sample data and the effect of merge were filtered by quality control to obtain the effective sequence of each sample. The non-repetitive sequences were clustered at a 97% similarity level to obtain operational taxonomic units (OTU). Each species was compared with the OTU database using the search representative software, and then the OTU was used to classify each species. After classification, OTU abundance was obtained according to the number of sequences in each OTU. Rumen bacterial composition of samples was determined by species annotation and abundance analysis, and further alpha diversity analysis was conducted to determine the differences among samples. Binformatic analysis of the OTU data was conducted through the Meiji biological cloud platform (http://login.majorbio.com/, accessed on 6 November 2020) provided by Shanghai Meiji Biotechnology Co., Ltd. (Shanghai, China) to determine the relative abundance, microbial diversity matrices, and other parameters.

### 2.8. Statistical Analysis

For each experimental run, the average of five fermentation bottles was taken that served as the experimental unit for statistical analysis. Data were analyzed by the analysis of variance (ANOVA) using a general linear model in SPSS software (SPSS, 2008). Statistical significance was declared at *p* < 0.05. The Alpha diversity index was calculated by Mothur software. The microbial Beta diversity was determined through (non-metric) multi-dimensional scaling (NDMS) of the Bray-Curtis dissimilarity matrix. Samples were grouped by treatment. PERMANOVA amongst all groups was performed (using 999 permutations). The linear discriminant analysis (LDA) effect size (LEfSe) was used to identify predominant bacterial taxa in each treatment group that can be considered biomarker taxa. In the present study, bacterial taxa having LDA scores (log10) > 2.5 were considered significantly different. PICRUSt was used to predict the function of 16S rDNA sequences. Spearman’s rank correlation (r) analyses were performed with the vegan R package (version 3.2) to analyze the relationship between the relative abundance of bacterial genera with rumen fermentation and amino acid parameters. Correlation heatmaps were constructed using the complot R package. In the two-dimensional heat map, the change in defined color and its depth indicates the nature and strength of the correlation, respectively. Asterisk sign was used when the r value was greater than 0.1 and the *p* values were less than 0.05 (* 0.01 < *p* ≤ 0.05, ** 0.001 < *p* ≤ 0.01, *** *p* ≤ 0.001).

## 3. Results

### 3.1. Total Gas Production, CH_4_ Production and Hydrogen Balance

**Figure 1:**
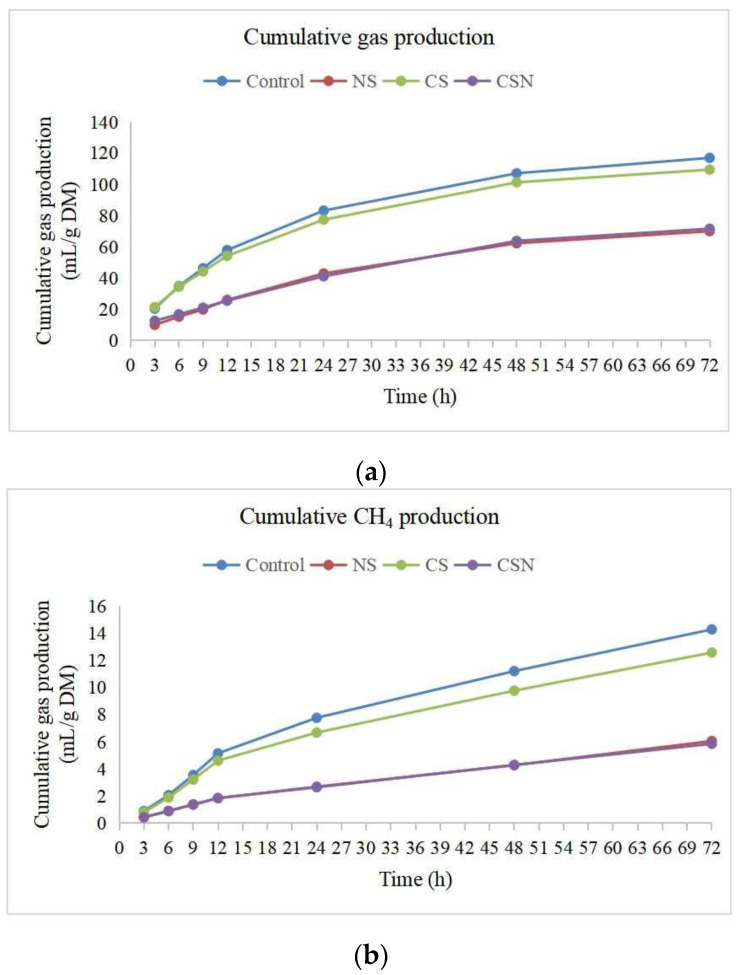
Cumulative gas production (**a**) and cumulative CH_4_ production (**b**) at different time intervals.

Total gas production, CH_4_ production, H_2_ produced, H_2_ utilized and H_2_ recovery in the treatment groups except CS were significantly lower than the control group (*p* < 0.05) (Table 2). The results showed that the cumulative gas production increased steadily from 3 h to 12 h, then slowly increased at 48 h. After that, a very low increase in gas production was observed. The cumulative gas production of SN and CSN was lower than that of the control group and CS group at different time intervals (Figure 1a). However, in the control group and CS, cumulative CH_4_ production showed an initial sharp increase within 12 h, followed by a stable continuous increase within 72 h (Figure 1b). However, the cumulative CH_4_ production of SN and CSN hardly changed within 12 h and then started to increase steadily at a much slower rate than that of the control group and CS. Moreover, the cumulative CH_4_ production curves of the SN and CSN were similar.

### 3.2. Rumen Fermentation Parameters

Cysteamine didn’t affect all fermentation parameters except acetate/propionate ratio (A/P ratio) which increased than the control group (Table 3). The SN and CSN showed higher pH, acetate, and MCP than the control group and CS (*p* < 0.05). The MCP concentration of the CSN was the highest (5.65). The concentrations of propionate, butyrate, isobutyrate, valerate, isovalerate, and TVFA in SN and CSN were lower than those in the control group and CS (*p* = 0.001). Treatment increased (*p* = 0.001) the A/P ratio as the highest (2.31) A/P ratio was observed for CSN followed by SN (2.20) and CS (1.86) as compared to the control. However, the treatment showed no effect on NH_3_-N and DMD (*p* > 0.05).

### 3.3. Ruminal Amino Acids

Treatment affected all ruminal amino acids except tyrosine and cysteine which did not exhibit any change (Table 4). The concentrations of total amino acids, total non-essential amino acids, total and individual essential amino acids (including leucine, methionine, threonine, phenylalanine, tryptophan, and isoleucine, excluding lysine and valine) were higher in SN and CS as compared to CSN and control group (*p* < 0.05). Treatment increased the valine as compared to the control revealing the highest value in CSN followed by SN and CS (*p* < 0.05). The concentration of lysine was lower in CS and CSN as compared to the control group (*p* < 0.05). Compared with the control group, the concentration of total non-essential amino acids in SN and CS increased, while decreased (*p* < 0.05) in CSN. The concentrations of alanine, glycine, glutamate, and asparagine were higher in SN and CS as compared to CSN and the control group (*p* < 0.05). Treatment significantly reduced the concentrations of histidine, arginine, glutamine, proline, and aspartic acid compared with the control group (*p* < 0.05).

### 3.4. Rumen Microbial Populations

The effect of treatment on rumen microbes is presented in Table 5. The total bacterial populations of CSN and SN were significantly higher than those of control and CS groups (*p* = 0.001). The total fungi population of CSN was significantly higher than those of control and CS (*p* = 0.009), and the methanogens in CSN was significantly higher than those of other groups (*p* = 0.001). However, there was no significant difference in the population of protozoa among the treatment groups (*p* = 0.168).

### 3.5. Rumen Bacterial Diversity

#### 3.5.1. Alpha and Beta Diversity Analysis

**Figure 2:**
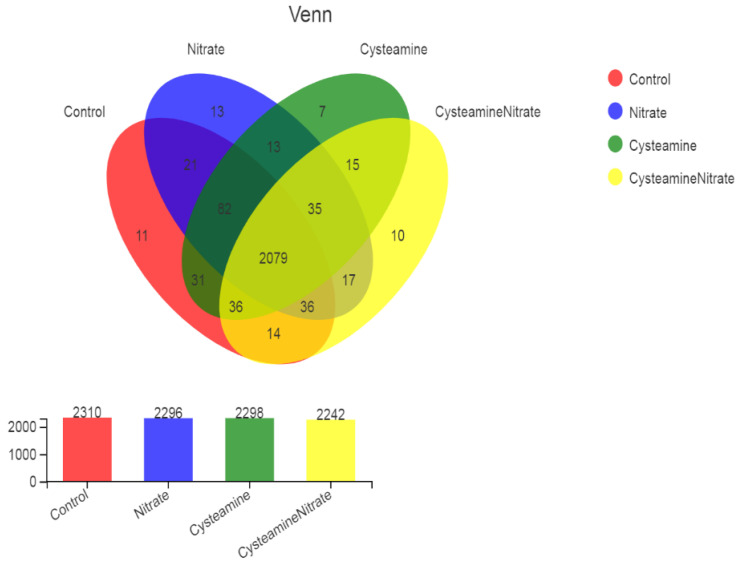
OTU distribution across different treatment groups.

Based on the sequence similarity (>97%), 2420 OTUs were obtained, belonging to 20 phyla, 38 classes, 79 orders, 139 families, 280 genera, and 554 species. The highest number of OTUs was found in the control followed by CS, SN, and CSN, respectively (Figure 2). The majority of OTUs (2079) were shared among four groups. The highest number of unique OTUs (13) was observed in SN followed by control (11), CSN (10), and CS (7).

The Coverage index for sequencing analysis was greater than 0.98, (Table 6), indicating the capturing optimum sequencing depth to reflect the real situation of microbial species in buffalo rumen fluid. Compared with CS and the control group, the Simpson index in SN and CSN increased, while the Shannon index decreased (*p* = 0.001). Compared with the control group, Ace, and Chao indexes were not affected in SN and CS but decreased significantly in CSN (*p* < 0.05).

**Figure 3:**
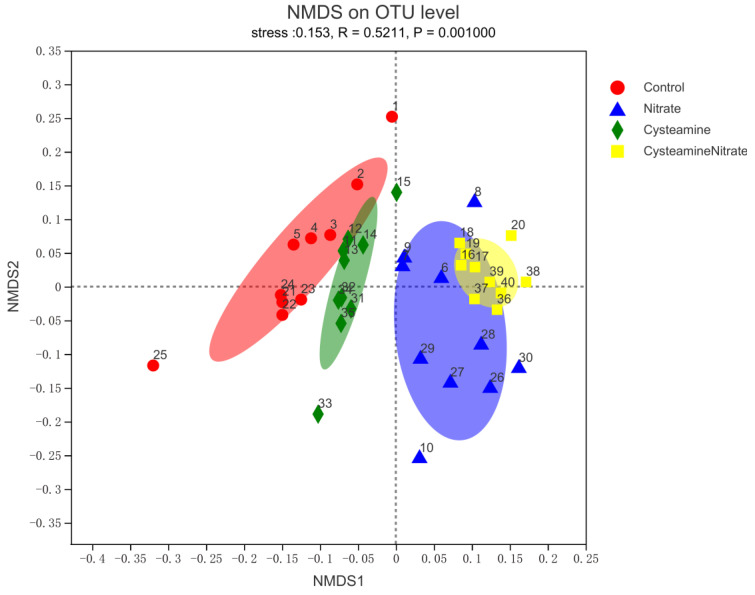
First two dimensions from the (non-metric) multi-dimensional scaling of the Bray-Curtis dissimilarity matrix. Samples were grouped by treatment. PERMANOVA amongst all groups (using 999 permutations) was performed (*p* = 0.001).

Beta diversity was determined through (non-metric) multi-dimensional scaling (NDMS) of the Bray-Curtis dissimilarity matrix using PERMANOVA with 9999 permutations which showed a significant effect (*p* = 0.001) of treatment (Figure 3). The results showed some differences in the composition of rumen flora between the treatment and control groups, while there was a certain similarity between the SN and CSN, but the individual difference was significant within the group.

#### 3.5.2. Relative Abundance of Bacterial Populations

**Figure 4:**
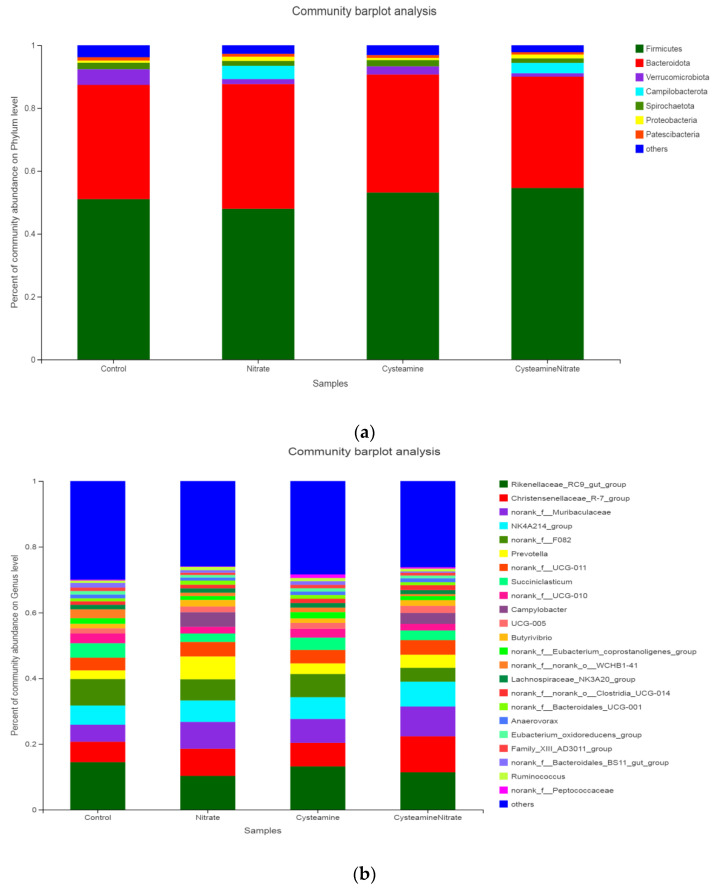
Relative abundance of rumen microflora of buffalo at phylum level (**a**) and genus level (**b**).

The relative abundance of microorganisms in the rumen contents of buffalo at phylum and genus levels is shown in Figure 4. The Firmicutes and Bacteroidota were dominant phyla that accounted for more than 85% of the whole rumen bacteriome (Figure 4a). Other major bacterial phyla were Verrucomicrobia, Campilobacterota, Spirochaetae, Proteobacteria, and Patescibacteria. The relative abundance of Firmicutes in SN was reduced (*p* = 0.001) compared with other groups (Table 7). However, no difference in the relative abundance of Bacteroidota among the groups was observed (*p* = 0.379). Treatment decreased (*p* = 0.001) the relative abundance of Verrucomicrobiota as compared to the control and CSN groups. Compared with the CS and control group, the relative abundances of Campilobacterota and Proteobacteria were increased in SN and CSN, but the relative abundance of Spirochaetota was decreased in SN and CSN (*p* = 0.001). The relative abundance of Patescibacteria was lower in SN and CSN as compared to the control group (*p* = 0.031).

The relative abundance of major bacterial genera was shown in Figure 4b. Rikenellaceae_RC9_gut_group was the dominant genus with the highest relative abundance among the four groups, and the secondary dominant genera were all less than 11%, including Christensenellaceae_R-7_group, norank_f__Muribaculaceae, NK4A214_group, norank_f__F082, *Prevotella*, norank_f__UCG-011, and *Succiniclasticum*, etc. Compared with the CS and control group, the relative abundances of Rikenellaceae_RC9_gut_group, norank_f__UCG-010 and norank_f__Eubacterium_coprostanoligenes_group were decreased in SN and CSN, but the relative abundances of Christensenellaceae_R-7_group, *Campylobacter*, and *Butyrivibrio* were increased (*p* = 0.001) in SN and CSN (Table 7). Treatment increased (*p* < 0.05) the relative abundances of norank_f__Muribaculaceae and *Prevotella* as compared to the control, however, decreased the relative abundances of *Succiniclasticum* and norank_f__norank_o__WCHB1-41 (*p* < 0.05). The relative abundances of NK4A214_group and UCG-005 of CSN were higher than those of other groups (*p* < 0.05). The relative abundance of norank_f__F082 was lower in CSN as compared to the control group (*p* = 0.001). The Lachnospiraceae_NK3A20_group was lower in CSN as compared to CS (*p* = 0.030).

#### 3.5.3. Biomarker Bacteria Taxa and Metagenomic Functional Profile

**Figure 5:**
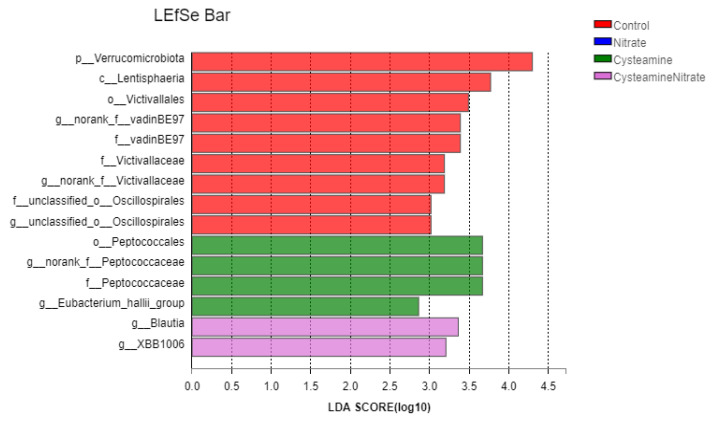
Biomarker bacterial genera in different treatment groups as revealed by linear discriminant analysis (LDA) Effect Size (LEfSe) based analysis (LDA score > 2.5).

**Figure 6:**
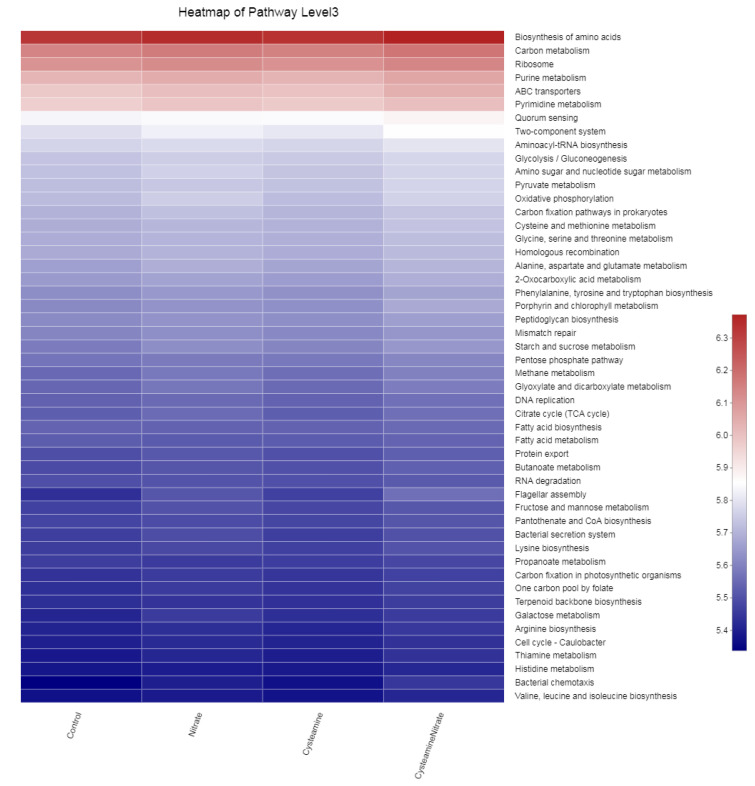
Top kyoto encyclopedia of genes and genomes (KEGG) enriched pathways in different treatment groups.

We identified bacterial taxa that were predominantly abundant as biomarkers among the treatment groups through LEfSe. A total of 15 significant taxonomic clades (LDA score > 2.5) were identified with 7 genera biomarkers (Figure 5). Two biomarker taxa including Peptococcaeae and Eubacterium hallii were identified as biomarkers in the CS group. However, highly selected bacterial genera in the CSN group were *Blautia* and XBB1006. Three genera, namely norank_f__vadinBE97, norank_f__Victivallaceae and unclassified_o__Oscillospirales were highly affected in control group. Metagenomic functional prediction revealed 50 enriched KEGG pathways as shown in Figure 6. The three most abundant pathways included the biosynthesis of amino acids, carbon metabolism, and the ribosome.

#### 3.5.4. Association of Rumen Bacteria with Ruminal Gas, Fermentation Parameters and Amino Acid Contents

**Figure 7:**
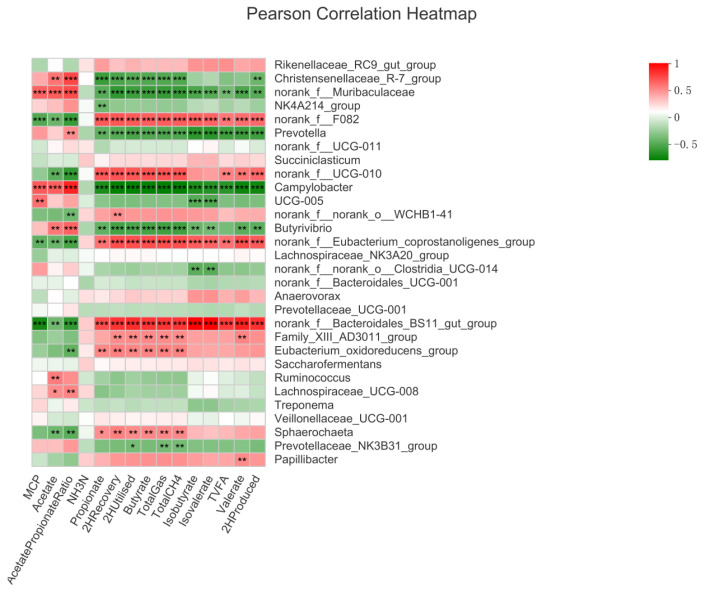
Correlation of bacterial genera with ruminal gas, hydrogen balance and rumen fermentation parameters. In the two-dimensional heat map, the change in defined color and its depth indicates the nature and strength of the correlation, respectively. Asterisk sign was used when the r value was greater than 0.1 and the *p* values were less than 0.05 (* 0.01 < *p* ≤ 0.05, ** 0.001 < *p* ≤ 0.01, *** *p* ≤ 0.001).

Our findings revealed three bacterial genera (norank_f__Muribaculaceae, *Prevotella* and *Campylobacter*) showed negative correlation (*p* < 0.01, r > 0.5) with gas, CH_4_, ruminal hydrogen balance (H_2_ produced, utilized, and recovery), total and individual VFA including propionate, butyrate, isobutyrate, isovalerate, and valerate, but three bacterial genera (norank_f__F082, norank_f__Eubacterium_coprostanoligenes_group and norank_f__Bacteroidales_BS11_gut_group) showed positive correlation (*p* < 0.01, r > 0.5) with these parameters (Figure 7). Three bacterial genera (norank_f__F082, norank_f__Eubacterium_coprostanoligenes_group and norank_f__Bacteroidales_BS11_gut_group) showed negative while Norank_f__Muribaculaceae and *Campylobacter* showed positive correlation with MCP, acetate and the A/P ratio (*p* < 0.01, r > 0.5). Christensenellaceae_R-7_group showed a positive correlation with acetate and A/P ratio but negatively correlated with gas, CH_4_, ruminal hydrogen balance (H_2_ produced, utilized, and recovery), propionate, and butyrate (*p* < 0.01, r > 0.5). The NK4A214_group showed negative correlation with propionate content (*p* < 0.01, r > 0.5). The *Prevotella* showed a positive correlation with the A/P ratio (*p* < 0.01, r > 0.5). Norank_f__UCG-010 was negatively correlated with acetate and A/P ratio but positively correlated with gas, CH_4_, ruminal hydrogen balance (H_2_ produced, utilized, and recovery), propionate, butyrate, valerate, and TVFA (*p* < 0.01, r > 0.5). The UCG-005 showed a negatively correlated with isobutyrate and isovalerate but a positively correlated with MCP (*p* < 0.01, r > 0.5). Norank_f__norank_o__WCHB1-41 showed negatively correlated with A/P ratio but positively correlated with H_2_ recovery (*p* < 0.01, r > 0.5). *Butyrivibrio* was positively correlated with acetate and A/P ratio but negatively correlated with gas, CH_4_, ruminal hydrogen balance (H_2_ produced, utilized, and recovery), and VFAs including propionate, butyrate, isobutyrate, isovalerate, and valerate (*p* < 0.01, r > 0.5). Norank_f__norank_o__Clostridia_UCG-014 showed negative correlation with isobutyrate and isovalerate (*p* < 0.01, r > 0.5). Family_XIII_AD3011_group positively correlated with gas, CH_4_, ruminal hydrogen balance (except H_2_ produced), butyrate, and valerate (*p* < 0.01, r > 0.5). Eubacterium_oxidoreducens_group was negatively correlated with the A/P ratio but positively correlated with gas, CH_4_, ruminal hydrogen balance (except H_2_ produced), propionate, and butyrate (*p* < 0.01, r > 0.5). *Ruminococcus* showed a positive correlation with acetate content (*p* < 0.01, r > 0.5). Lachnospiraceae_UCG-008 was positively correlated with acetate and A/P ratio (*p* < 0.05, r > 0.5). Sphaerochaeta was negatively correlated with acetate and A/P ratio but positively correlated with gas, CH_4_, ruminal hydrogen balance (except H_2_ produced), propionate, and butyrate (*p* < 0.05, r > 0.5). *Prevotella* ceae_NK3B31_group showed a negative correlation with gas, CH_4_, and H_2_ recovery (*p* < 0.05, r > 0.5). Papillibacter showed positive correlation with valerate (*p* < 0.01, r > 0.5).

**Figure 8:**
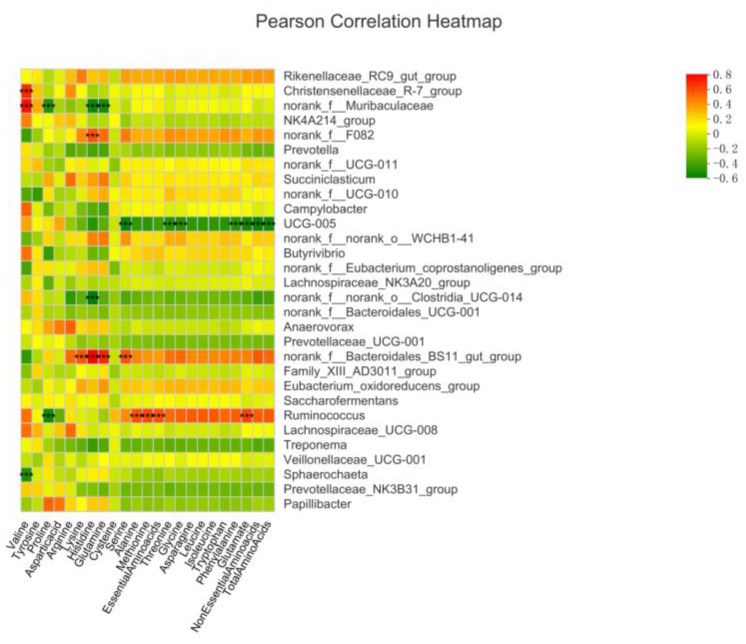
Correlation of bacterial genera with ruminal amino acid contents. In the two-dimensional heat map, the change in defined color and its depth indicates the nature and strength of the correlation, respectively. Asterisk sign was used when the r value was greater than 0.1 and the *p* values were less than 0.05 (* 0.01 < *p* ≤ 0.05, ** 0.001 < *p* ≤ 0.01, *** *p* ≤ 0.001).

Spearman’s correlation between the relative abundance of bacterial genera and ruminal amino acid contents is shown in Figure 8. Christensenellaceae_R-7_group was a positively correlated with valine, but Sphaerochaeta showed positive correlation (*p* < 0.001, r > 0.5). Norank_f__Muribaculaceae was positively correlated with valine but negatively correlated with proline, glutamine, and cysteine (*p* < 0.001, r > 0.5). The Norank_f__F082 showed positively correlation with Histidine (*p* < 0.001, r > 0.5). The UCG-005 showed a negative correlation with serine, threonine, glycine, phenylalanine, glutamate, non-essential amino acids, and total amino acids (*p* < 0.001, r > 0.5). Norank_f__Bacteroidales_BS11_gut_group was positively correlated with lysine, histidine, glutamine, and serine (*p* < 0.001, r > 0.5). *Ruminococcus* showed a negative correlation with proline but positively correlated with alanine, methionine, essential amino acids, and glutamate (*p* < 0.001, r > 0.5).

## 4. Discussion

### 4.1. Total Gas Production, CH_4_ Production and Hydrogen Balance

The results of the present study showed that the addition of SN alone and the mixture of SN and CS significantly reduced the total gas volume, CH_4_ production, H_2_ produced, H_2_ utilized, and H_2_ recovery. Cysteamine alone did not affect these indicators. It showed that SN was the main factor affecting CH_4_ formation and hydrogen balance. Our findings are consistent with earlier studies that reported the negative effects of nitrates on CH_4_ production in vitro [19,20,21,22,23,24]. In vivo studies have also reported that long-term supplementation of coated nitrate can continuously reduce the intestinal CH_4_ emissions of grazing steers [25]. Studies have shown that nitrate can not only direct the metabolic H_2_ away from CH_4_ production, but also reduce the relative abundance of H_2_-producing Firmicutes [26]. The results of this study also supported this mechanism. Sodium nitrate significantly reduced the production, utilization, and recovery of H_2_, and also reduced the relative abundance of Firmicutes.

### 4.2. Rumen Fermentation Parameters

Rumen VFA is an important energy source for ruminants. In the present study, the addition of SN alone and the mixture of SN and CS significantly increased the ruminal pH, but it was within the normal range of rumen pH value (6.0~7.5). The rumen pH value is affected by the balance between ammonia, VFA production, and lactate. The addition of SN in the present study reduced the concentrations of butyrate, isobutyrate, valerate, isovalerate, and TVFA. These findings are consistent with previous studies reporting supplementation of sodium nitrate under in vitro cultures [22,24,27,28,29,30]. The reason for the increase in rumen pH value added with nitrate may be related to the decrease of VFA content in rumen fluid [31]. The decrease of VFA content in rumen fluid is mainly attributed to the toxic effect of nitrate on rumen bacteria and its strong inhibitory effect on in vitro rumen fermentation [32,33]. In the present study, nitrate increased the A/P ratio, which is consistent with the findings of earlier studies [3,27,34]. This is mainly because SN promotes the growth of nitrate-reducing bacteria and consumes hydrogen and VFA. Thermodynamically, the reduction process of nitrate is easier than the formation of propionic acid [35]. The reduction process of nitrate competes for hydrogen electrons with the formation of CH_4_ and propionate, leading to the reduction in CH_4_ and propionate production. Acetate in the rumen mainly comes from the fermentation of cellulose and hemicellulose by microorganisms, and this process is accompanied by the production of hydrogen. The reduction process of nitrate consumes oxygen, alleviates the inhibition of hydrogen on fiber degradation, and promotes the production of acetate, which is also one of the reasons why nitrate can change the mode of rumen fermentation and increases the A/P ratio [36].

Many studies [5,37,38,39,40] have shown that in a low-protein diet, nitrate supplementation can be used as an NPN source, and can even effectively replace other NPN sources (such as urea) to promote the synthesis of MCP in the rumen. The affinity between hydrogen and nitrate is higher than that with CO_2_. Therefore, when nitrate is present in the rumen, hydrogen preferentially combines with nitrate to produce ammonia nitrogen and reduces the production of CH_4_ [35]. This process can not only effectively reduce CH_4_ production, but also provides raw materials for the synthesis of microbial proteins. It has been reported that [9,41] cysteamine can change the rumen internal environment, improve the ability of microorganisms to use ammonia nitrogen, and is conducive to the synthesis of microbial protein. In the present study, we found that the addition of sodium nitrate and cysteamine can enhance the synthesis of rumen MCP. Cysteamine supplementation alone had no effect on the content of NH_3_-N and MCP, which is inconsistent with the previous results of in vitro studies [9] and in vivo studies [8]. Some studies [42] revealed that cysteamine can improve the synthesis of VFA, and sufficient energy supply in the rumen can enhance the activity of microorganisms and speed up the utilization of NH_3_-N. At the same time, the relative balance of energy and nitrogen in the rumen is also an important factor to improve the synthesis of MCP. Nitrate supplementation has shown different effects on NH_3_ concentration under in vitro conditions [28,29,32], which indicates that nitrate reduction to ammonia is not the only way of nitrate metabolism in rumen [43].

Studies have shown that adding nitrate to ruminant diets already adapted to nitrate can promote nutrient DMD [44]. It is reported that adding cysteamine to the diets of pigs, cattle, sheep, and poultry has a positive impact on production performance, while the impact on dry matter intake and DMD was usually very small [45]. However, studies have also shown that adding cysteamine can improve the degradation rate of NDF [8]. In the present study, the addition of sodium nitrate and cysteamine did not affect DMD. The reasons for the different results may be related to the different dosages of sodium nitrate and cysteamine or the different in vivo and in vitro test conditions.

### 4.3. Ruminal Amino Acids

The amino acids in rumen fluid come from the degradation of dietary protein and endogenous amino acids. In the present study, the results revealed that the addition of sodium nitrate and cysteamine alone had a positive effect on the content of total amino acids, while the addition of their mixture exhibited a negative effect on the content of total non-essential amino acids. Previous in vitro studies [28] have shown that the addition of 1% nitrate significantly increased the total and individual AA contents except for cysteine. In vivo studies showed that feeding 70 g/d sodium nitrate to buffalo can increase the contents of total and most individuals AA, and have no negative effect on amino acids in rumen fluid [46]. The effect of sodium nitrate on the content of amino acids in the present study is different from earlier studies. Nitrate can inhibit methanogenesis in rumen batch culture, but not all H can turn into microbial amino acid biosynthesis as a substitute for CH_4_ [47]. Cysteamine, as a somatostatin inhibitor, can enhance the body’s protein synthesis and metabolism, and increase the deposition of nitrogen, which has a significant growth-promoting effect [45,48]. However, the effect of Cysteamine on rumen amino acids is rarely reported, therefore further in vivo studies are required to corroborate these findings.

### 4.4. Rumen Microbial Populations

Studies have shown that nitrate inhibits CH_4_ production while increasing the number of total bacteria and methanogens [28]. However, treatment with 1 mg/mL nitrate under in vitro conditions, did not affect the number of methanogens while increasing the total number of bacteria but the number of methanogens decreased with supplementation of 3 mg/mL nitrate. Interesting, nitrate at a dose rate of 1–3 mg/mL could inhibit CH_4_ production [49]. Therefore, the number of methanogens cannot directly determine the CH_4_ production. In the present study, the addition of 1 mg/mL nitrate alone increased the total number of bacteria but did not affect the number of methanogens, fungi, and protozoa, but reduced CH_4_ production. These findings might be attributed to the type of substrate and dose of sodium nitrate because higher concentrations of nitrate are toxic to rumen microorganisms, hence an appropriate dose of sodium nitrate can be used as a non-protein nitrogen source to provide nitrogen for rumen microorganisms [3]. Moreover, appropriate nitrate levels have shown to increase the relative abundance of rumen bacteria [24,30], especially the nitrate-reducing bacteria [50].

Studies have shown that cysteamine can affect the characteristics of biofilm, and change the process of H_2_ circulation in the rumen and the microecological environment [51]. The decrease of rumen NH_3_-N in response to cysteamine supplementation is consistent with the decrease of rumen protozoa [52]. In vitro studies have shown that cysteamine directly affects the rumen microbiota by decreasing the number of rumen protozoa while increasing the accumulation of hydrogen [53]. Furthermore, cysteamine can inhibit the growth of protozoa or parasitic bacteria [54], but the results are not consistent regarding the effect of cysteamine on the inhibition of protozoa in the rumen. Studies have reported that cysteamine significantly decreased the number of protozoa when added at a low dose, but significantly increased the number of protozoa in rumen fluid when added at a dose of 30 g/D [8]. The supplementation of cysteamine in the present study exhibited no effect on the number of rumen protozoa, mainly because of the use of a lower dose of cysteamine which did not affect the protozoa population. The present study also revealed that cysteamine alone did not affect the number of total bacteria, fungi, and methanogens, which was consistent with the previous studies [8]. However, our study also revealed that the mixture of sodium nitrate and cysteamine is favorable for the synthesis of microbial protein and increases the number of rumen microorganisms.

### 4.5. Rumen Bacterial Diversity

The alpha diversity analysis of buffalo rumen flora in this experiment showed that the coverage of each group was higher than 98%, indicating that the sequencing results truly reflected the species and structural diversity of the buffalo rumen bacterial community. Sodium nitrate reduced the diversity of rumen bacteriome (Shannon index and Simpson index) in the present study which is in agreement with earlier studies [28]. In the present study, the mixture of sodium nitrate and cysteamine reduced the diversity and richness of buffalo rumen flora. Studies have shown that nitrate supplementation in ruminants can shift the composition of rumen bacterial communities [21] owing to nitrite toxicity (a nitrate reduction pathway intermediate), and by creating competition for hydrogen and changing the ruminal pH [24,55]. Earlier studies have shown that cysteamine does not affect the alpha diversity of buffalo rumen microbiota [56]. Beta diversity was affected by sodium nitrate and cysteamine in the present study which is consistent with earlier reports [50,57].

The Firmicutes and Bacteroidota were major bacterial phyla observed in buffalo rumen in the present study, which is consistent with previous studies [50,58]. In the present study, sodium nitrate alone reduced the relative abundance of Firmicutes which is consistent with earlier studies describing the reduction of essential branched chain VFAs contents by inhibiting the growth of cellulose-degrading microbes [59]. Verrucomicrobiota can produce short-chain fatty acids, through the digestion of polysaccharides such as cellulose [60]. In the present study, the relative abundance of Verrucomicrobiota decreased significantly after the addition of sodium nitrate and cysteamine, suggesting that the addition of sodium nitrate and cysteamine might be associated with the reduction of short-chain fatty acids. Spirochaetae can degrade cellulose, hemicellulose, pectin, etc., which has an important impact on the conversion of plant fibrous substances to VFA [61]. In the present study, sodium nitrate and cysteamine reduced the relative abundance of Spirochaetae, indicating that sodium nitrate may inhibit the degradation of cellulosic substances by rumen microorganisms, which also explains the reason for the significant reduction of the TVFAs contents in response to nitrate treatment. Campilobacterota contains many species with nitrate reductase genes [62], which is crucial for the optimal utilization of nitrate. Our study revealed an increase in the relative abundance of Campilobacterota in response to the supplementation of sodium nitrate and cysteamine, which is in agreement with earlier studies [20,28]. The increase in Campilobacterota can avoid nitrite accumulation and subsequent toxic effects on the rumen ecosystem.

The relative abundance of Rikenellaceae is positively correlated with the feed utilization rate of the host and the metabolism of VFAs and short-chain fatty acids [63,64]. Our study found that the addition of sodium nitrate and cysteamine alone and in combination reduced the relative abundance of Rikenellaceae_RC9_ gut_group while decreasing the concentrations of butyrate, isobutyrate, valerate, isovalerate, and total volatile fatty acids, revealing the negative impact on VFA synthesis. Christensenellaceae belongs to the phylum Firmicutes, which mainly decomposes fibrous substances to produce acetic acid. Our study found that the addition of sodium nitrate and cysteamine increased the relative abundance of christensenellaceae_ R-7_group, along with acetate content. Muribaculaceae and *Prevotella* belong to Bacteroidota, which participate in the metabolism of a variety of microorganisms and can degrade hemicellulose with high activity [65]. On the other hand, *Prevotella* is one of the main H_2_ utilization bacteria, and its abundance increases under the condition of CH_4_ inhibition [18]. Our study revealed that sodium nitrate and cysteamine can increase the relative abundance of Muribaculaceae and *Prevotella*. The combination of sodium nitrate and cysteamine inhibits CH_4_ production, indicating the decomposition and utilization of non-cellulosic material in buffalo rumen, and nitrate can stimulate H_2_ to inhibit CH_4_ production by using bacterial growth. *Succiniclasticum* can metabolize succinic acid produced by rumen microorganisms after decomposing carbohydrates into propionate, and then produce propionate [66]. In the current study, the relative abundance of *Succiniclasticum* in rumen fluid decreased significantly after treatment, which may be one of the reasons for the significant reduction of propionate concentration. *Campylobacter* is a gram-negative non-fermenting bacterium containing nitrate reductase genes [62]. *Butyrivibrio* is a bacterium that decomposes and utilizes cellulose in the rumen [67], and it can use hydrogen [68]. Our study revealed that sodium nitrate and cysteamine can increase the relative abundance of *Campylobacter* and *Butyrivibrio* while inhibiting methanogenesis at the same time. It indicates that sodium nitrate can have a positive effect on nitrate-reducing bacteria and hydrogen-utilization bacteria, which facilitates to reduce the nitrite accumulation in the rumen during nitrate reduction, and avoids nitrite toxicity.

### 4.6. Biomarker Bacteria Taxa and Metagenomic Functional Profile

The Peptococcaeae and Eubacterium hallii were identified as biomarkers of the cysteamine group in the rumen in the present study. Peptococcaeae can use peptones or amino acids as energy sources to produce VFAs [69]. Eubacterium hallii is an important intestinal bacterium responsible for metabolism and producing short-chain fatty acids [70]. Our study revealed that the cysteamine group had a higher abundance of Peptococcaeae and Eubacterium halli, indicating that cysteamine favored the synthesis of short-chain fatty acids and had a positive impact on rumen fermentation.

Our findings revealed that the dominant bacterial genera in the CSN group were *Blautia* and XBB1006. Studies have shown that *Blautia* can promote the biosynthesis of rumen lysine and branched-chain amino acids, and promote the breaking of carbohydrate ester bonds leading to better degradation and utilization of polysaccharides [71]. Moreover, *Blautia* is positively correlated with the content of short-chain fatty acids (acetate, propionate, butyrate), which is desirable for the decomposition of non-digestible carbohydrates [72]. Our study also indicated that the combination of cysteamine and sodium nitrate is conducive to the synthesis of microbial protein, and improves acetate and A/P.

Metagenomic functional prediction revealed 50 enriched KEGG pathways including biosynthesis of amino acids, carbon metabolism, and ribosome with the highest abundance. No substantial change in the top three pathways in different treatment groups reveals the functional redundancy of the microbial ecosystem as these pathways did not significantly differ despite substantial changes observed in the relative abundance of rumen bacteria.

### 4.7. Association of Rumen Bacteria with Ruminal Gas, Fermentation Parameters and Amino Acid Contents

Our results showed that fibrolytic bacteria positively correlated with gas, CH_4_, ruminal hydrogen balance (H_2_ produced, utilized, and recovery), and VFAs (including propionate, butyrate, isobutyrate, valerate, isovalerate, and TVFA, which is consistent with earlier studies [28]. Studies have shown that the cluster containing fibrobacilli positively correlated with CH_4_ as it provides a substrate for methanogenesis and is compared with other bacteria and fungi [73]. In addition, carbohydrates are the main substrate for the production of VFAs in rumen. That’s why fiber-degrading bacteria are positively correlated with the production of CH_4_ and VFA, and is also the main reason for the decrease of VFA content when we inhibit methanogenesis. Our findings revealed that *Prevotella* and *Complylobacter* showed a negative association with ruminal gas, CH_4_, hydrogen balance (H_2_ produced, utilized, and recovery), and VFAs (including propionate, butyrate, isobutyrate, valerate, isovalerate and TVFA), which agrees with earlier studies [28]. *Prevotella* is mainly responsible for the utilization of H_2_ [26]. Methane emission depends on the abundance of H_2_-producing bacteria [74]. A corollary to this is the observation that chemical inhibition of methanogenesis in goats led to increases in the abundance of H_2_-consuming *Prevotella* spp. [18].

Our findings revealed that Christensenellaceae_R-7_group showed a positive correlation with valine, Norank_f__Bacteroidales_BS11_gut_group showed a positive correlation with lysine, histidine, glutamine, and serine, while *Ruminococcus* was positively correlated with alanine, methionine, Essential Amino acids, and glutamate. Christensenellaceae is a cellulose-decomposing bacterium. Bacteroidales can use cellulose, xylan, arabinogalactan, and pectin, as well as plant starch to obtain energy [58]. *Ruminococcus* can degrade cellulose, hemicellulose, and lignin in roughage in the rumen to produce acetate [75]. These bacteria are cellulose-degrading bacteria, which contribute towards the synthesis of amino acids. Studies have shown that low protein feed increases the abundance of fibrolytic bacteria, leading to enhanced cellulytic activity, and synthesis of microbial AA and protein [28]. This is a major reason that fibrolytic bacteria positively correlated with rumen AA content in the present study. Further in vivo studies are required to corroborate these findings and improve the nutrient digestibility in buffalo.

## 5. Conclusions

Our study revealed that cysteamine had no effect on CH_4_ production and rumen fermentation parameters except the A/P ratio, but nitrate significantly reduced the cumulative gas and CH_4_ production. Cysteamine combined with nitrate significantly increased the proportion of microbial protein, A/P ratio, and the number of total bacteria, fungi, and methanogens, while reducing the cumulative gas and CH_4_ production. Nitrate and cysteamine alone significantly increased the contents of total non-essential and essential amino acids. Biomarker taxa for Cysteamine included Peptococcaeae and Eubacterium hallii, while *Blautia* and XBB1006 were the most dominant genera in the nitrate and cysteamine mixture group. Our findings concluded that the combination of Cysteamine and nitrate was favorable for the synthesis of microbial protein, and rumen microorganisms, but same it also reduced the CH_4_ and total gas production which is nutritionally beneficial.

## Figures and Tables

**Table 1:** Major ingredients and chemical composition of the experimental basal feed substrate based on dry matter (DM).

Ingredient	Content
Elephant grass (%)	90.0
Concentrate Mixture (%) *	10.0
Chemical composition	
Dry matter (%)	20.0
Crude protein (%)	9.07
Neutral detergent fiber (%)	75.3
Acid detergent fiber (%)	46.0
Ash (%)	9.72
Gross Energy (kcal/kg DM)	4.69

* Concentrate mixture (corn 17.83%, wheat bran 7.51%, soybean meal 5.72%, limestone 0.5%, CaHPO_4_ 0.6%, NaHCO_3_ 0.8%, NaCl 0.7%, Premix1 0.34%). The additive premix provided the diet with the following (per kg of diet): VA 550,000 IU, VE 3000 IU, VD3 150,000 IU, 4.0 g Fe (as ferrous sulfate), 1.3 g Cu (as copper sulfate), 3.0 g Mn (as manganese sulfate), 6.0 g Zn (as zinc sulfate), 80 mg Co (as cobalt sulfate).

**Table 2:** Effects of sodium nitrate (SN), cysteamine hydrochloride(CS), and combination of CS and SN (CSN) supplementation on in vitro ruminal batch culture total gas production, methane emission, and reductive hydrogen (0–72 h).

Items	Control	SN	CS	CSN	SEM	*p* Value
Total gas production (mL/gDM)	117 ^a^	69.9 ^b^	120 ^a^	71.5 ^b^	1.85	0.001
Methane production (CH_4_, mL/gDM)	14.3 ^a^	6.05 ^b^	13.8 ^a^	5.84 ^b^	0.23	0.001
Reductive hydrogen						
H_2_ Produced (mmol)	9.89 ^a^	8.46 ^b^	9.98 ^a^	8.49 ^b^	0.11	0.001
H_2_ Utilized (mmol)	8.10 ^a^	5.51 ^b^	8.06 ^a^	5.38 ^b^	0.08	0.001
H_2_ Recovery (%)	81.9 ^a^	65.1 ^b^	80.8 ^a^	63.3 ^b^	0.29	0.002

Values with different superscripts in the same row differ signifificantly.

**Table 3:** Effects of sodium nitrate (SN), cysteamine hydrochloride(CS), and combination of CS and SN (CSN) supplementation on in vitro ruminal fermentation fermentation parameters.

Items	Control	SN	CS	CSN	SEM	*p* Value
pH	6.83 ^b^	6.90 ^a^	6.81 ^b^	6.89 ^a^	0.01	0.001
Acetate (mmol/L)	38.3 ^b^	42.0 ^a^	39.2 ^b^	42.9 ^a^	0.46	0.001
Propionate (mmol/L)	21.4 ^a^	19.1 ^b^	21.1 ^a^	18.6 ^b^	0.24	0.001
Butyrate (mmol/L)	13.4 ^a^	6.94 ^b^	13.45 ^a^	6.75 ^b^	0.13	0.001
Isobutyrate (mmol/L)	2.05 ^a^	1.60 ^b^	1.99 ^a^	1.53 ^b^	0.03	0.001
Valerate (mmol/L)	2.13 ^a^	1.61 ^b^	2.16 ^a^	1.62 ^b^	0.03	0.001
Isovalerate (mmol/L)	4.47 ^a^	3.54 ^b^	4.36 ^a^	3.43 ^b^	0.06	0.001
Total volatile fatty acid (TVFA, mmol/L)	81.7 ^a^	74.8 ^b^	82.3 ^a^	74.9 ^b^	0.99	0.001
Acetate to propionate ratio (A/P)	1.79 ^d^	2.20 ^b^	1.86 ^c^	2.31 ^a^	0.01	0.001
Ammonia nitrogen (NH_3_-N, mg/100 mL)	18.7	18.1	18.8	18.5	0.19	0.109
Microbial protein (MCP, mg/mL)	4.01 ^c^	4.82 ^b^	4.06 ^c^	5.65 ^a^	0.15	0.001
Dry matter digestibility (DMD, %)	43.0	39.8	42.0	37.0	1.92	0.173

Values with different superscripts in the same row differ signifificantly.

**Table 4:** Effects of sodium nitrate (SN), cysteamine hydrochloride(CS), and CS and SN (CSN) supplementation on in vitro ruminal batch culture amino acid profile (ng/mL).

Items	Control	SN	CS	CSN	SEM	*p* Value
Alanine	131 ^c^	486 ^a^	321 ^b^	149 ^c^	38.3	0.001
Valine	236 ^d^	384 ^b^	286 ^c^	460 ^a^	23.2	0.001
Histidine	1119 ^a^	759 ^b^	639 ^c^	378 ^d^	69.3	0.001
Arginine	289 ^a^	197 ^b^	126 ^c^	223 ^b^	16.2	0.001
Glycine	102 ^c^	226 ^a^	161 ^b^	98.2 ^c^	14.2	0.001
Glutamine	57.4 ^a^	16.7 ^b^	18.9 ^b^	10.1 ^b^	4.84	0.001
Glutamate	1690 ^c^	3851 ^a^	3160 ^b^	1849 ^c^	233	0.001
Proline	95.7 ^a^	29.4 ^c^	44.6 ^bc^	49.2 ^b^	6.55	0.001
Leucine	45.7 ^c^	214 ^a^	145 ^b^	60.2 ^c^	18.2	0.001
Lysine	40.9 ^a^	28.4 ^ab^	13.4 ^b^	11.6 ^b^	3.83	0.006
Methionine	22.5 ^c^	148 ^a^	101 ^b^	44.2 ^c^	13.0	0.001
Tryptophan	26.9 ^c^	105 ^a^	60.6 ^b^	25.4 ^c^	8.56	0.001
Phenylalanine	68.3 ^c^	314 ^a^	195 ^b^	69.5 ^c^	27.0	0.001
Threonine	125 ^c^	281 ^a^	205 ^b^	107 ^c^	19.1	0.001
Isoleucine	40.7 ^c^	209 ^a^	140 ^b^	55.2 ^c^	18.2	0.001
Tyrosine	35.0	36.1	36.2	40.9	1.51	0.519
Serine	291 ^b^	436 ^a^	295 ^b^	204 ^c^	24.2	0.001
Asparagine	44.0 ^c^	204 ^a^	141 ^b^	59.7 ^c^	7.05	0.001
Aspartic acid	20.9 ^a^	11.8 ^b^	8.91 ^b^	12.5 ^b^	1.44	0.001
Cysteine	4.00	4.38	4.96	5.40	0.47	0.757
Essential Amino acids ^1^	607 ^c^	1685 ^a^	1146 ^b^	833 ^c^	108.	0.001
Non-Essential Amino acids ^2^	3880 ^c^	6258 ^a^	4955 ^b^	3079 ^d^	318	0.001
Total Amino Acids	4487 ^c^	7943 ^a^	6101 ^b^	3912 ^c^	418	0.001

Values with different superscripts in the same row differ signifificantly. ^1^ isoleucine, leucine, lysine, methionine, phenylalanine, threonine, tryptophan, valine; ^2^ Histidine, Alanine, arginine, glycine, glutamine, glutamate, proline, tyrosine, serine, aspartic acid, asparagine, cysteine.

**Table 5:** Effects of sodium nitrate (SN), cysteamine hydrochloride(CS), and combination of CS and SN (CSN) supplementation on microbial populations (log10 copies per g of rumen contents).

Items	Control	SN	CS	CSN	SEM	*p* Value
Bacteria	11.9 ^c^	12.1 ^ab^	12.0 ^bc^	12.3 ^a^	0.03	0.001
Fungi	9.97 ^b^	10.2 ^ab^	10.1 ^b^	10.4 ^a^	0.04	0.009
Protozoa	8.13	8.55	8.54	8.52	0.08	0.168
Methanogens	10.0 ^b^	10.1 ^b^	10.1 ^b^	10.3 ^a^	0.03	0.001

Values with different superscripts in the same row differ signifificantly.

**Table 6:** Effects of sodium nitrate (SN), cysteamine hydrochloride(CS), and CS and SN (CSN) supplementation on bacterial alpha diversity parameters.

Items	Control	SN	CS	CSN	SEM	*p* Value
Shannon index	6.07 ^a^	5.85 ^b^	6.01 ^a^	5.73 ^b^	0.03	0.001
Shimposon index (×10^−2^)	0.70 ^b^	1.05 ^a^	0.77 ^b^	1.11 ^a^	0.05	0.001
Ace index (×10^3^)	1.90 ^a^	1.90 ^a^	1.90 ^a^	1.82 ^b^	0.01	0.001
Chao index (×10^3^)	1.92 ^a^	1.93 ^a^	1.93 ^a^	1.84 ^b^	0.01	0.031
Coverage (%)	98.8	98.7	98.7	98.8	0.02	0.941

Values with different superscripts in the same row differ signifificantly.

**Table 7:** Effects of sodium nitrate (SN), cysteamine hydrochloride(CS), and combination of CS and SN (CSN) supplementation on relative abundance of different bacteria phyla and genera (%).

Taxonomic Level	Microbes	Control	SN	CS	CSN	SEM	*p* Value
Phylum	Firmicutes	50.9 ^a^	46.8 ^b^	52.5 ^a^	54.3 ^a^	4.02	0.001
	Bacteroidota	36.2	40.0	37.8	35.8	3.73	0.379
	Verrucomicrobiota	5.13 ^a^	2.16 ^b^	2.72 ^b^	1.03 ^c^	1.34	0.001
	Campilobacterota	0.02 ^b^	4.33 ^a^	0.01 ^b^	3.18 ^a^	0.96	0.001
	Spirochaetota	2.02 ^a^	1.59 ^b^	2.24 ^a^	1.49 ^b^	0.64	0.021
	Proteobacteria	0.69 ^b^	1.34 ^a^	0.70 ^b^	1.21 ^a^	0.35	0.001
	Patescibacteria	1.01 ^a^	0.90 ^ab^	0.86 ^ab^	0.76 ^b^	0.21	0.031
Genus	Rikenellaceae_RC9_gut_group	13.4 ^a^	11.3 ^b^	12.6 ^a^	11.2 ^b^	1.95	0.005
	Christensenellaceae_R-7_group	6.94 ^c^	7.61 ^b^	7.18 ^c^	10.57 ^a^	1.64	0.001
	norank_f__Muribaculaceae	4.75 ^c^	7.71 ^b^	7.18 ^b^	9.04 ^a^	1.56	0.001
	NK4A214_group	6.21 ^bc^	5.94 ^c^	6.61 ^b^	7.44 ^a^	1.04	0.005
	norank_f__F082	7.54 ^a^	6.07 ^ab^	6.51 ^ab^	4.15 ^b^	0.85	0.001
	*Prevotella*	2.62 ^d^	6.63 ^a^	3.31 ^c^	4.40 ^b^	2.10	0.019
	norank_f__UCG-011	4.04	4.16	4.41	4.34	1.07	0.540
	*Succiniclasticum*	4.65 ^a^	3.00 ^b^	3.16 ^b^	3.40 ^b^	1.92	0.027
	norank_f__UCG-010	2.84 ^a^	1.98 ^b^	2.75 ^a^	1.93 ^b^	0.38	0.001
	*Campylobacter*	0.01 ^b^	4.32 ^a^	0.01 ^b^	3.15 ^a^	0.95	0.001
	UCG-005	1.68 ^b^	1.63 ^b^	1.75 ^b^	2.12 ^a^	0.37	0.031
	norank_f__norank_o__WCHB1-41	3.21 ^a^	1.46 ^b^	1.40 ^b^	0.61 ^c^	1.13	0.001
	*Butyrivibrio*	1.24 ^b^	1.99 ^a^	1.45 ^ab^	1.73 ^a^	0.33	0.001
	norank_f__Eubacterium_coprostanoligenes_group	1.68 ^a^	1.08 ^b^	1.89 ^a^	1.21 ^b^	0.36	0.001
	Lachnospiraceae_NK3A20_group	1.23 ^ab^	1.37 ^ab^	1.56 ^a^	1.20 ^b^	0.31	0.030

Values with different superscripts in the same row differ signifificantly.

## Data Availability

The sequence data generated in this experiment (16SrRNA genesequences) were deposited in SRA database of NCBI under Bioproject No. PRJNA865458 and SRA accession No. SRP389551.
